# Supplementary material for: A Computational Analysis Based on Automatic Digitization of Movement Tracks Reveals the Altered Diurnal Behavior of the Western Flower Thrips, Frankliniella occidentalis, Suppressed in PKG Expression
Source: Insects. 2025 Mar 19;16(3):320. doi: 10.3390/insects16030320 (PMC11943175; doi:10.3390/insects16030320)
Supplement: Supplementary file 1 [file insects-16-00320-s001.zip › insects-3487551-supplementary.pdf]

insects

MDPI

Supplementary Information

Table S1. Primers used in this study and their PCR conditions

Genes	Primers (5'→3')	Annealing temperature (℃)	Product size (bp)
**Fo-PKG-qPCR-F1**	ACCCAATGAAGACCTACAAC	55	200
**Fo-PKG-qPCR-R1**	TCAGTCACATTGTGTACGTT		
**Fo-dsPKG-F1**	TAATACGACTCACTATAGGGAAGATCTCCCA	55	320
**Fo-dsPKG-R1**	TAATACGACTCACTATAGGGCACCATGTC		
**Fo-CLK-F1**	CAACAGTGATCCTCTTTGGA	55	200
**Fo-CLK-R1**	TCCTTCATTGGGTTCAGATG		
**Fo-Per-F1**	CAATGTAAGTTTCAGCGTCC	53	210
**Fo-Per-R1**	AGTAGCTACCTTCATCACCA		

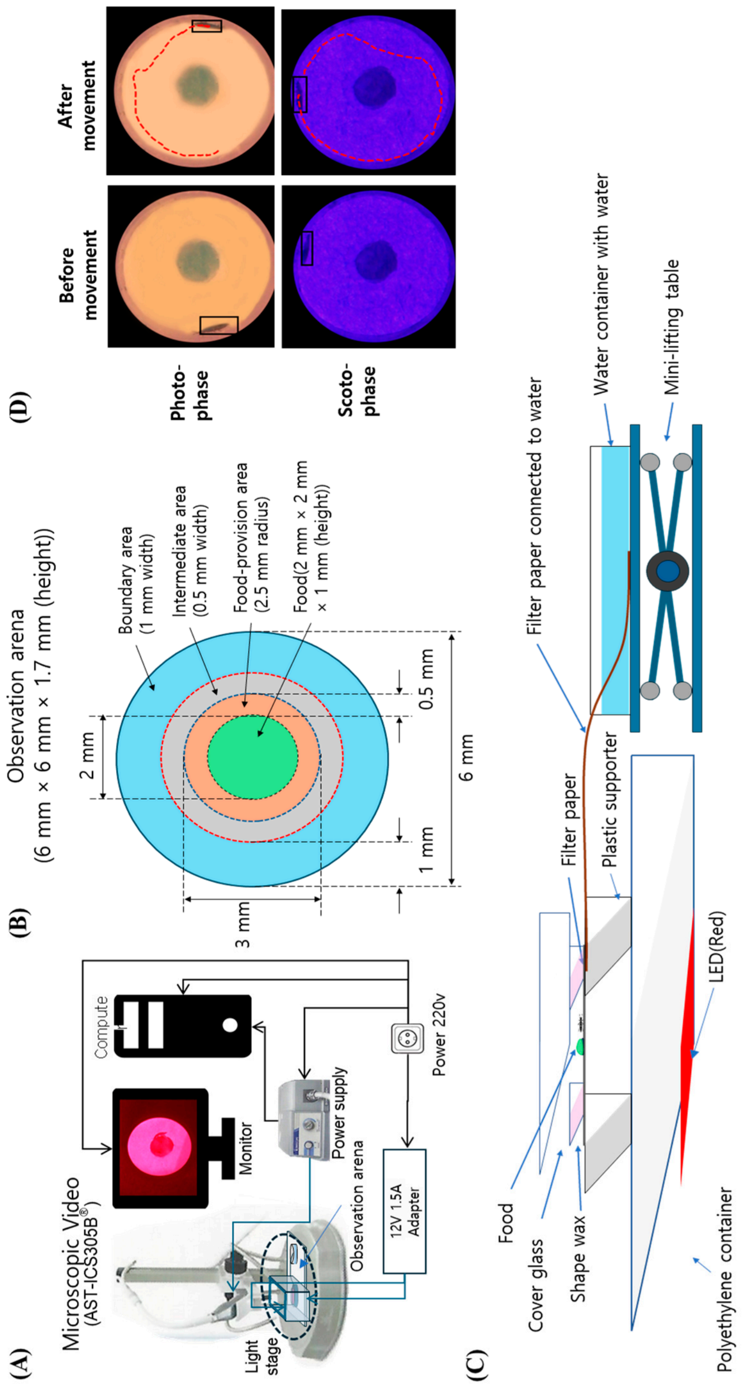

Figure S1. Observation of continuous movement of *F. occidentalis* under the conditions described below. (A) Observation system. (B) Micro-areas in the observation arena (top view). (C) Observation arena with food and water supply (side view). (D) An example of movement tracks in photo- and scoto-phases.

[Behavior-monitoring conditions]

Individual adult female thrips at 2-3 days after emergence were observed continuously for 24 h in each trial. The observation system was installed on an optic fiber microscope (Camscope®, AST-ICS305B, Sometech Vision, Seoul, Korea) consisting of observation arena, camera, computer, and light (Fig. S1A). The observation arena (6 mm in diameter and 1.7 mm in depth) (Fig. S1B) was made of dental modelling wax modified from Akinyemi et al. (2021). A hole (6 mm in diameter) was produced in the middle of ring-shaped

dental wax (20 mm in diameter and 1.7 mm in depth) for serving as the movement stage for thrips. In the middle of the movement stage, a particle (2 mm in diameter and 1 mm in height) of fresh bean just after germination was provided as food. To provide water to the food during the observation period, a strip of filter paper (2 × 90 mm) was used for connecting the filter paper, which was placed underneath the observation arena, to a reservoir of water (dechlorinated tap water; 10 mL) located outside the observation arena (Fig. S1C). The food, with the provision of water, was sufficiently fresh for feeding for the observation period of one day. The observation arena was divided into the food-provision (2.5 mm radius), edge (1 mm width), and intermediate (0.5 mm width) areas (Fig. S1B).The observation arena, covered with a piece of glass (34 × 34 × 5 mm (height)) on the top and attached with a filter paper (20 × 20 mm) at the bottom, was placed over a plastic supporter (42 × 42 × 10 mm (height)), and again laid on a white polyethylene container (white) with a cover (100 × 100 × 10 mm (height)) (Fig. S1C). At the bottom of the container, a layer of LED (Red; 12V, 1.5 A) was located to serve as a light source for the red light in scotophase. To provide the light for photophase, an external light stage framed with transparent polyethylene (110 × 80 × 5 mm (width) for each wall), was placed over the observation arena (see dotted ellipse in Fig. S1A). Over the light stage, two LED (White; 12V, 1.5A) bars (100 × 70 × 2 mm (height)) were installed above the walls.

(A)

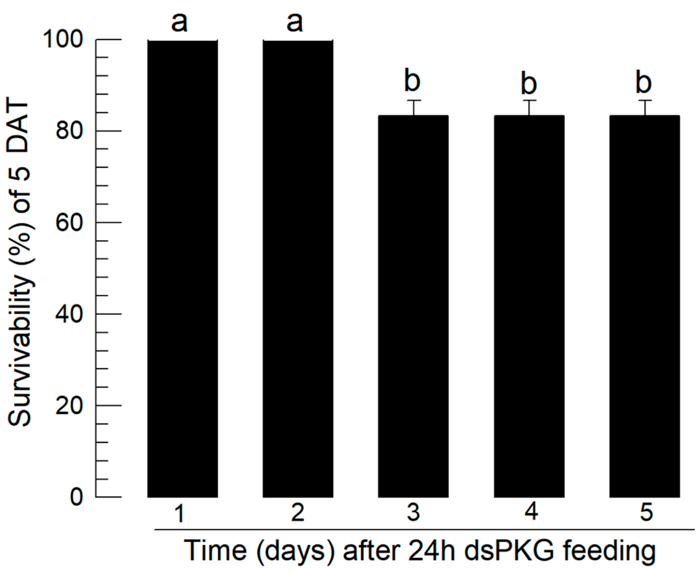

(B)

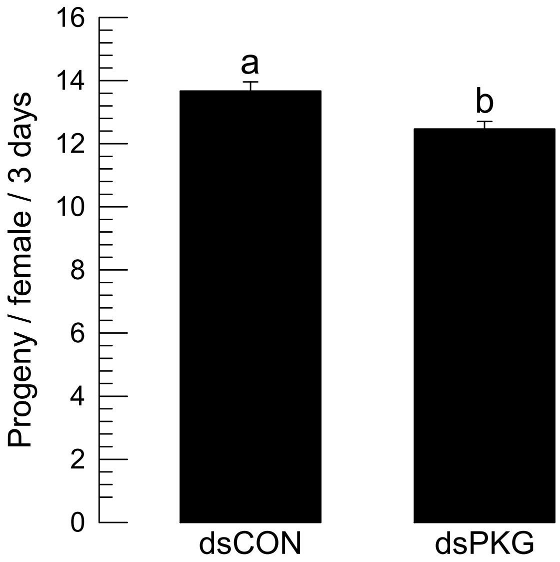

Figure S2. Influence of *PKG* suppression by its specific RNAi on immature development (A) and adult fecundity (B) in *F. occidentalis*. Test thrips were fed with dsRNA specific to PKG ('dsPKG') at 500 μg/mL for 24 h. For dsCON, a nontarget gene, *EGFP*, was used for RNAi dsCON ('dsCON'). Each treatment was replicated three times. Different letters above standard deviation bars indicate significant differences among means at Type I error = 0.05 (LSD test).
